# Rare diseases: What rheumatologists need to know?

**DOI:** 10.1186/s42358-024-00407-6

**Published:** 2024-09-27

**Authors:** Renan Rodrigues Neves Ribeiro do Nascimento, Daniela Gerent Petry Piotto, Eutilia Andrade Medeiros Freire, Fabricio de Souza Neves, Flavio Roberto Sztajnbok, Blanca Elena Rios Gomes Bica, Frederico Augusto Gurgel Pinheiro, Katia Tomie Kozu, Ivanio Alves Pereira, Valderilio Feijo Azevedo, Rafael Alves Cordeiro, Henrique Ayres Mayrink Giardini, Marco Túlio Muniz Franco, Margarida de Fátima Fernandes Carvalho, Nilton Salles Rosa-Neto, Sandro Félix Perazzio

**Affiliations:** 1 Universidade Federal de Sao Paulo - Escola Paulista de Medicina, Rua Botucatu, 740, 3º andar, São Paulo, SP 04023-062 Brazil; 2 Paraiba Federal University (Universidade Federal da Paraiba), João Pessoa, Brazil; 3 Federal University of Santa Catarina (Universidade Federal de Santa Catarina), Florianópolis, Brazil; 4 Federal University of Rio de Janeiro (Universidade Federal do Rio de Janeiro), Rio de Janeiro, Brazil; 5 USP FM (Universidade de Sao Paulo Faculdade de Medicina), Pacaembu, Brazil; 6 Unisul (Universidade do Sul de Santa Catarina), Tubarão, Brazil; 7 Federal University of Parana (Universidade Federal do Parana), Curitiba, Brazil; 8 Federal University of Amapá (Universidade Federal do Amapa), Macapá, Brazil; 9 State University of Londrina (Universidade Estadual de Londrina), Londrina, Brazil; 10 UNISA (Universidade de Santo Amaro), São Paulo, Brazil; 11 Fleury Laboratories, Av. Morumbi, 8860, Sao Paulo, SP 04580-060 Brazil

**Keywords:** Rare diseases, Orphan diseases, Rheumatic diseases

## Abstract

Although the terms “rare diseases” (RD) and “orphan diseases” (OD) are often used interchangeably, specific nuances in definitions should be noted to avoid misconception. RD are characterized by a low prevalence within the population, whereas OD are those inadequately recognized or even neglected by the medical community and drug companies. Despite their rarity, as our ability on discovering novel clinical phenotypes and improving diagnostic tools expand, RD will continue posing a real challenge for rheumatologists. Over the last decade, there has been a growing interest on elucidating mechanisms of rare autoimmune and autoinflammatory rheumatic diseases, allowing a better understanding of the role played by immune dysregulation on granulomatous, histiocytic, and hypereosinophilic disorders, just to name a few. This initiative enabled the rise of innovative targeted therapies for rheumatic RD. In this review, we explore the state-of-the art of rare RD and the critical role played by rheumatologists in healthcare. We also describe the challenges rheumatologists may face in the coming decades.

## Introduction

Over the past five centuries, interest in the approach of disorders with low prevalence has slowly increased. The concept of rare diseases (RD) is discussed as far as 1581 by Rembert Dodoens, a Flemish physician author of “*Medicinalium Observationum Exempla Rara*,* Recognita et Aucta*”, in which an extensive list of 200 RD is described [1]. However, over the centuries, the distinction between RD and orphan diseases (OD) remains unclear and may have different definitions.

The National Institute for Health Orphan Drug Act has defined RD as a disease or condition that affects fewer than 200,000 people in the United States in 1983, representing a prevalence of 86 cases/100,000 citizens at that time [2]. European Union currently considers RD those affecting fewer than 1 in 2000 citizens (or 50 in 100,000 people), and the World Health Organization (WHO) suggested a frequency of less than 65 per 100,000 people [3]. Although the terms RD and OD are often used interchangeably, OD are defined as those underappreciated or neglected by the medical community and drug companies, for which research on diagnosis and effective treatment is sparse. Therefore, RD and OD are not synonymous, as some low-income countries prevalent diseases without commercial interest of research may still be orphan [4]. Although controversial, the National Institute for Health and Care Excellence for Drugs has introduced a novel subcategory of ultra-rare diseases, representing any illness with a prevalence lower than 1 per 50,000 individuals [5].

Despite the increasing burden of RD over time, reliable epidemiologic data are still lacking [6]. A total of 7000 RD affecting 20.5 million people in the United States and 46.5 million in Europe have been identified. Although individually rare, they collectively affect almost 10% of the global population, and this number is continuously growing as an estimated 250 new RD are being described yearly [5, 6]. Although awareness of RD has increased over the last decades, underdiagnosis is still a reality, and a long avenue to improve the unmet demands of the area is open. In this review, we explore the field of RD and how rheumatologists can play a critical role in their healthcare.

## Epidemiological aspects

Although literature is laconic on unquestionable studies determining the real prevalence of RD worldwide, Wakap et al. [7] recently estimated that 3.5–5.9% of the world’s population (263–446 million people) might carry any RD. As 71.9% of these diseases have a genetic etiology and, therefore commonly manifest early in life, 69.9% of them are exclusive to the pediatric age group. Nevertheless, genetic inheritance also contributes to the understanding that certain rare diseases are more prevalent in smaller ethnicities or populations with a high rate of consanguinity (e.g., Gaucher disease and cystic fibrosis in Ashkenazi Jews), or in other subpopulations where the presence of a specific condition represents a biological survival advantage against other environmental challenges (e.g., sickle cell anemia in regions of Africa with a high prevalence of malaria) [7].

Moreover, up to 80.7% of these studied patients were concentrated in only 4.2% of the 6000 considered RD, all of which had a prevalence of 10–50/100,000 individuals, thus falling into the more “common” epidemiological stratum within the realm of reality [7]. Could this discovery truly be attributed to the higher frequency of these diseases? Or is it secondary to a greater number of diagnoses provided by physicians, given that, unlike lesser-known conditions, these clinical entities are at least discussed during their medical training? Despite the absence of a definite answer for this question, the financial burden of having a rare disease in the United States is significant and has a direct impact on public health. According to the National Organization for Rare Disorders (NORD) 2019 survey, 76% of respondents reported experiencing financial challenges due to their own or their family member’s rare diagnosis. Furthermore, certain support resources are not being fully utilized due to the lack of clinical data on unmet financial resources, direct and indirect costs of treatments, patient disability, or lack of benefits for caregivers [8].

## Policy panorama and main limitations for RD research

Since its creation in 1983, the Orphan Drug Act has designated over 5000 drugs and biologics for OD in the United States [9]. Despite considerable progress in product approvals, the majority of the estimated 7000 known RD remains unaddressed [10]. Analysis of an internal Food and Drug Administration (FDA) database reveals that only 19% of the 199 drugs designated for OD from 1983 to 2019 have been approved [9].

Several caveats on RD-related scientific research challenges new orphan drugs approval and clinical trials conduction, including (but not restricted to): (1) unclear pathophysiology mechanisms, which make it difficult to identify potential therapeutic targets; (2) limited patient population, hardening recognition and recruitment of individuals; (3) heterogeneous manifestations, making it difficult to homogenize populational samples; (4) lack of well-established diagnostic criteria and reliable biomarkers for robust statistical data; (5) higher prevalence in children, which challenges ethics committees approval; (6) post-marketing safety concerns; and (7) lack of follow-up metrics and geographic dispersion of RD patients, which preclude regular blood draws and sequential evaluations in studies [11].

Approximately one in 13 people currently live with an undiagnosed condition, which makes them prone to being overlooked. The Undiagnosed Diseases Network (UDN) was created in 2014 to improve the level of diagnosis and care of undiagnosed diseases, facilitate research, and create an integrated and collaborative database community to improve patient management. The UDN was funded by the National Institutes of Health (NIH) and aims to solve unsolved cases worldwide (https://undiagnosed.hms.harvard.edu) [12]. The integration of novel technologies, including Ribonucleic acid (RNA) sequencing, long-read sequencing, and periodic reanalysis of whole exome sequencing (WES) and whole genome sequencing (WGS), has played a crucial role in driving advancements in diagnostic rates in UDN [13].

In Brazil, the Ministry of Health issued Ordinance 199 on January 30, 2014, aiming to reduce morbidity and mortality while enhancing the quality of life for patients with RD by instituting the National Policy for Comprehensive Care of People with Rare Diseases [14]. This initiative laid the groundwork for the establishment of the Brazilian Rare Diseases Network (RARAS-BRDN), comprised of 40 voluntary reference institutions across the country. Although data in this field are still emerging in Brazil, this group published in 2022 the first epidemiological survey of diagnoses for 13 rare diseases, providing a reliable estimate based on the Brazilian public health database: approximately 55,000 individuals. However, extrapolating Wakap et al. [7] results, the estimate of Brazilian RD patients would be approximately 7.2–12.2 million [7, 15]. Despite the remarkable work conducted by the authors, it is numerically evident that there remains a tremendous challenge ahead to identify and support millions of other affected individuals.

In December 2020, Brazilian interministerial committee was initiated by decree 10,558, tasked with the formulation, coordination, and execution of projects, policies, programs, and initiatives directed at safeguarding of RD patients [16]. Physical and mental well-being, as well as advancing and defending the rights of individuals with RD were also encompassed. Concurrently, in September 2023, the Mixed Parliamentary Front for Innovation and Technologies in Rare Diseases emerged, comprising senators and deputies. Additionally, analogous efforts at the state level, exemplified by the Legislative Chamber of the Federal District, share a common purpose: deliberating, advocating, and proposing measures to enhance the support for RD patients. On December 29, 2023, the official gov.br website reported the recommendation by the National Committee for Health Technology Incorporation (CONITEC) for the assimilation of technologies tailored to RD. These assimilations serve as pivotal components in shaping public healthcare policies and are integral for potential inclusion within the National Agency of Supplementary Health (ANS) coverage, thereby influencing forthcoming healthcare programs. The website publication delineates explicit recommendations, encompassing interventions such as Trikafta^®^ for cystic fibrosis, agalsidase beta for Fabry disease, neonatal screening, and others. This underscores an elevated receptiveness of CONITEC towards incorporating state-of-the-art medical technologies since the establishment of the interministerial committee [16].

Another pivotal policy against disinformation regarding RD is to dedicate a day in the year to raise awareness for rare diseases. Since 2008, Rare Disease Day has been commemorated on February 29, precisely because it is a “rare” date. In non-leap years, this day is celebrated on February 28, yet maintaining its purpose: a time to celebrate progress in research and advocacy efforts, and to reflect on ongoing challenges (https://www.rarediseaseday.org/) [17].

## Rheumatologist as a bridge for RD

Patients with RD often face a “diagnostic odyssey” from the onset of the disease, resulting in an unbetterable therapeutic delay [18, 19]. In 2019, NORD found that only 36% of patients with RD were diagnosed within the first year, and 28% reported a delay of seven or more years, with 38% being misdiagnosed during their diagnostic journey [20, 21]. Rheumatologists routinely evaluate patients who have already been seen by multiple specialists and may play an important role on their “diagnostic odyssey.” Moreover, the area experienced a critical growth lately, as rheumatologists are increasingly adopting previously considered OD, such as Mikulicz syndrome and Riedel thyroiditis, which are now recognized fibroinflammatory conditions under the umbrella of IgG4-related disease (IgG4-RD) [22]. Therefore, IgG4-RD was compared to “a black crow flying through the dark night” as it passed unscathed upon the history of rheumatic diseases.

In addition, broad spectrum gene sequencing assays overcame unsolved rheumatic cases into novel RD, such as monogenic forms of relapsing polychondritis (VEXAS Syndrome - vacuoles, E1 enzyme, X-linked, autoinflammatory, somatic) or polyarteritis nodosa (deficiency of Adenosine deaminase 2) [21, 22]. Similarly, as the description of new inborn errors of immunity (IEI) exponentially increases, new entities previously classified as undifferentiated autoinflammatory diseases have been defined [23]. In the upcoming decades, the focus of the specialty should be on raising awareness of RD as a cornerstone of health care and research.

## Red flags for RD in rheumatology

Given the wide clinical spectrum of RD, it is difficult to develop general clues and warning signs involving all RD of interest to the rheumatologist. Jeffrey Modell Foundation’s warning signs for primary immunodeficiencies diagnosis may help the identification of underlying IEI in patients with rheumatic conditions, such as recurrent, severe, persistent, or unusual infections, especially those unexplained by predisposing factors such as asplenia, malignancies or immunosuppressive treatment. Maybe even more presumptive is the coexistence of polyautoimmunity, life-threatening or treatment refractory rheumatic disorders, mainly in younger ages, which may represent a relevant sign of an IEI [24, 25].

“Red flags” suggesting an IEI may also be considered among those patients presenting with uncommon associations of rheumatic conditions, such as systemic lupus erythematosus (SLE) and Behçet disease in A20 haploinsufficiency or relapsing polychondritis and polyarteritis nodosa in VEXAS. Noteworthy, rheumatologists should be also attentive whenever IEI-associated manifestations, such as autoimmune cytopenia, sarcoid-like inflammation, benign or malignant lymphoproliferative (among other malignancies) are concurrent to rheumatic manifestations. Last, but not least, given the monogenic basis of IEI, a family history of primary immunodeficiency or immune dysregulation should be definitely valued for inherited underlying conditions as the etiology of a rheumatic disease [24, 25].

In an effort to suggest clinical and laboratory criteria for RD and OD screening, the Brazilian Society of Rheumatology has established a task force: the Rare Diseases Committee (RDC). RDC aims to lead scientific research, provide support for patients, and promote information about RD to rheumatologists and healthcare professionals involved in this field. Based on their own experience, the experts suggested 9 clinical and 6 laboratorial “red flags” that should trigger the suspicion of RD in the context of rheumatic manifestations (Table 1).

When dealing with multiorgan unexplained features, rheumatologists should always rule out infection, malignancy, and classic autoimmune diseases before proceeding with RD investigation. Due to the vast number and heterogeneity of RD, 11 subgroups of the most significant conditions for the rheumatologist were proposed, including: (1) autoinflammatory diseases (Table 2); (2) other primary immune regulatory disorders (PIRD) (Table 3); (3) granulomatous diseases (Table 4); (4) histiocytosis disorders (Table 5); (5) primary connective tissue diseases (Table 6); (6) storage diseases (Table 7); (7) metabolic bone diseases (Table 8); (8) rare vasculopathies (Table 9); (9) hypereosinophilic syndromes (Table 10); (10) adjuvant-induced rheumatic diseases (Table 11); and (11) rare infectious/parasitic diseases (Table 12).

## Novel tools for investigating RD in rheumatology

Numerous advancements in RD approach have impacted rheumatological practices lately, necessitating ongoing education regarding newly identified autoantibodies, cytokine signatures, imaging modalities, and enhanced accessibility to molecular diagnoses [19]. Rheumatologists now encounter novel autoantibodies in the field of myositis, which have facilitated the proposition of updated diagnostic classifications based on a better understanding of the pathophysiology of these conditions and more precise recognition of clinical phenotypes and prognoses [26].

The description of a subgroup of patients with lupus manifestations and type 1 interferon signature, currently identified as interferonopathies, provided insights into the molecular pathways involved in systemic lupus erythematosus and opened a significant avenue for targeted anti-interferon therapies [27, 28]. 18 F-fluorodeoxyglucose-positron emission tomography/computed tomography has been progressively indicated for monitoring disease activity and assessing target organ damage at baseline. This promising tool has been applied to evaluate treatment responses and helps diagnosing multisystemic diseases such as sarcoidosis, IgG4-related disease, amyloidosis, and relapsing polychondritis [29, 30]. Whole-body magnetic resonance (WB-MR) imaging is now utilized in a multitude of rheumatic, bone, and muscle disorders, resulting in a robust and sophisticated tool for the detection, staging, and monitoring of various non-oncologic musculoskeletal conditions such as chronic recurrent multifocal osteomyelitis/chronic non-bacterial osteomyelitis (CRMO/CNO) [31, 32].

As a result of greater access to molecular diagnosis, an exponential increase in the discovery of new PIRD was observed in the last decade [23]. Interestingly, in the immune-mediated rheumatic diseases universe, different pathogenic variants in the same gene can lead to widely discordant phenotypes. As a result, identification of a defect in a PIRD-associated gene should be considered based on its immunologic function and not discounted solely on previously described phenotype with that gene. VEXAS syndrome is a good example, as it disrupts the paradigm by utilizing a genotype-first approach, where a large genetic database is analyzed for commonalities among patients with minimal consideration of clinical phenotypes [33, 34]. By broadening the phenotype, we have discovered that RD may be more prevalent than previously thought, with new evidence indicating that *Ubiquitin Like Modifier Activating Enzyme 1* (*UBA1*) pathogenic variants are present in 1 in 13,591 individuals [35].

Since 2010, the number of pathogenic variants discovered each year has increased rapidly, due to advances in Deoxyribonucleic acid (DNA) sequencing technologies and big data analysis. As of May 2017, Orphanet lists 3573 RD-linked genes. Many relatively low-cost phenotype-driven commercial and customizable gene sequencing panels are available. Exome sequencing has become more cost-effective, offering new opportunities for Mendelian disease diagnosis and research [36]. The field of multi-omics methodologies will transform disease discovery and diagnosis by utilizing advanced technologies beyond DNA sequencing, including metabolomics, epigenomics, RNA-Seq (transcriptomics), and proteomics [37].

Regrettably, several challenges are present in the “diagnostic odyssey” of patients with RD, and health policies must be implemented to encourage research. Additionally, more randomized controlled trials are urgently needed to address this significant unmet need [11]. A comprehensive understanding of current research efforts, knowledge/research gaps, and funding patterns is critical to systematically expedite the pace of research discovery in rare diseases [38].

## Targeted therapy for rheumatological RD

Despite some progress in the last decades, only 5% out of the 7000 identified RD have an approved treatment. Improvement of this therapeutic gap may be multifaceted challenge impeded by factors such as limited access to medical expertise, diagnostic testing, and translational approaches [39, 40]. According to NORD, while only 29% of the patients with any RD had been authorized to use off-label FDA unapproved treatment for their medical condition, 61% were denied or faced delays in obtaining therapies that necessitated pre-approval from an insurance company [20, 21]. As RD constitute a heterogeneous group of illnesses, numerous therapeutic methods have been currently established, including enzyme replacement therapies (ERT), oligonucleotide therapies, monoclonal antibodies (mAb), gene therapies, cell therapies, anti-fibrotic agents, amongst others [41].

ERT involve low molecular weight compounds that regulate biological processes, typically administered as an infusion of a native or recombinant enzyme. ERT have long been a fundamental element in treating RD associated with an enzyme loss of function, such as lysosomal storage disorders [40]. Approved examples of ERT are agalsidase β and agalsidase α for Fabry disease; laronidase for Mucopolysaccharidosis type 1 and alglucosidase α for Pompe disease [40].

Oligonucleotide therapies are a promising strategy for intervening at the RNA level. Antisense oligonucleotides (ASO) and small interfering ribonucleic acid (siRNA) are the most extensively studied and can both prevent specific disease-associated protein translation by promoting degradation of its mRNA [40]. In 2018, patisiran became the first siRNA FDA approved therapy, shortly followed by the approval of the ASO inotersen [42]. Both drugs act by degrading mRNA encoding transthyretin (TTR) protein, which reduces tissue TTR deposits and clinically improves neurological manifestations of familial transthyretin amyloidosis [40].

Immunotherapies with mAb have been employed since 1983, using antibodies to specifically block immune targets, resulting in signaling pathways modulation, cell recruitment to specific sites, cytotoxins delivery, or circulating factors neutralization. Some mAb, such as canakinumab and rilonacept, have been authorized to address IL-1β inflammasome release in cryopyrin-associated periodic syndromes and other autoinflammatory syndromes, modifying the natural history of these diseases [43, 44].

Gene therapy is a therapeutic approach that involves nucleic acids delivery in cell nuclei, with the aim of replacing defective genes or inserting normal copies into the genome. This modality holds promise for treating monogenic diseases (138). Notably, ex vivo transfection utilizing lentivirus vectors has been employed in pioneering clinical trials for primary immune deficiency diseases such as adenosine deaminase deficiency, severe combined immunodeficiency (SCID), and Wiskott-Aldrich syndrome [45–47].

Cell therapy was originally developed for cancer treatment, with the introduction of chimeric antigen receptor (CAR) T-cells as an immunotherapeutic modality to combat the disease. Despite its early stage of development, CAR T-cell therapy is being explored as a potential option for treating autoimmune disorders such as refractory systemic lupus erythematosus, with successful outcomes reported and few severe adverse events such as cytokine release syndrome [48].

Antifibrotic agents, such as nintedanib and pirfenidone, exert their therapeutic effects by inhibiting tyrosine kinase activity, thus impeding the progression among fibrosing interstitial lung diseases (ILD) [49, 50]. Nintedanib has been shown to be efficacious in autoimmune [51] and systemic sclerosis associated ILD by mitigating the rate of forced vital capacity decrease [52]. Nevertheless, extensive research is necessary to fully elucidate the true benefits of these agents in the context of these heterogenous diseases.

As the understanding of RD progressively grows and novel translational therapies become available, it is imperative to share efforts among non-profit socially motivated organizations and the pharmaceutical industry to develop and improve access to more affordable orphan drugs. While global discussion relies on the equalization of profitable development of novel therapies and the available fundings to finance a lifelong treatment for a small number of citizens in a society, this approach would benefit not only patients but also national healthcare systems [53].

## Non-governmental organizations and web-based resources in RD

As a multitude of domains within RD care remain unaddressed, therefore efforts to promote a reliable and comprehensible source of information through accessible platforms and tools are fundamental. Non-governmental organizations or philanthropic groups that advocate for a particular disease play a pivotal role in serving as a conduit to scientific medical societies or public and private resources. This comprehensive approach aims to overcome misperceptions and to shorten the “diagnostic odyssey” of individuals affected by RD [54].

Platforms like NORD (https://rarediseases.org/) and EURORDIS (European Organization for Rare Diseases - https://www.eurordis.org/) not only support patients and families affected by RD, but also guide patients, caregivers, healthcare professionals, and researchers in diagnosing and selecting specific treatments or participating in ongoing clinical trials for particular diseases [55, 56]. In Brazil, a similar initiative with information fully endorsed by the Brazilian Society of Medical Genetics (https://muitossomosraros.com.br) in partnership with a Federal Committee from the National Parliament (Mixed Parliamentary Front for Comprehensive Care for People with Rare Diseases) offers relevant content and facilitates patient access to reference centers.

As a crucial resource for genetic RD awareness, Online Mendelian Inheritance in Man (OMIM; http://www.omim.org/) provides a platform with a database of genetic syndromes, their phenotypes, inheritance patterns, and genetic variants, which helps physicians in determining diagnosis of RD all around the world. Additionally, Orphanet (http://www.orpha.net/) serves as a diagnostic aid and aims to disseminate information regarding novel treatment strategies available for RD [57]. Similarly, FindZebra (http://www.findzebra.com/) has been developed as an algorithm that employs freely available information to suggest suitable RD diagnoses for physicians and patients [58].

## Future perspectives

The “diagnostic odyssey” involving misdiagnoses, lack of specialists, and lack of access to an acceptable molecular diagnosis culminating in therapeutic delays, is an unmet demand that needs to be addressed in the upcoming decades. Artificial intelligence (AI) with deep learning technology holds great promise for RD. Big data software could gather various sources (multi-omics, patient registries, clinical trials) to overcome RD barriers (low diagnostic rates, reduced number of patients, geographical dispersion). AI is expected to create accessible algorithms able to integrate data among patients and health providers, improving diagnostic approaches, prognosis scoring, disease stratification, decision support, and health records [59–61].

Despite all the above-described efforts, there is no approved drug for 94% of all RD. A novel precision medicine-based approach can drive good discriminator assays designed to targeted therapy testing to predict its potential benefit individually. With a better understanding of molecular and environmental mechanisms causing RD, we will be able to deliver not only symptomatic treatment but also curative personalized medicine [53].

The challenges for the next decades are multiple, including case identification, medical education, investment in infrastructure, and optimization of financial resources for specific centers trained in RD. As a guide to improve the “diagnostic odyssey” of RD patients, European institutions and Member States have created a rare 2030 action campaign with eight recommendations: 1- a roadmap for RD long-term policies; 2- integrated European and national plans and strategies; 3- earlier, faster, and more accurate diagnosis; 4- access to high-quality healthcare; 5- integrated and person-centered care in partnership with patients; 6- innovative and need-led research development; 7- optimizing data for patient and societal benefit; 8- available, accessible, and affordable treatments [62].

## Conclusion

While individually rare, the aggregated number of individuals with RD or OD represents a significant health burden. Rheumatologists will increasingly face unsolved cases in the next decades and play a cornerstone role in RD healthcare. The specialty’s efforts should focus on promoting awareness that these diseases may be rare, but patients cannot be orphans. Recognizing zebras among the horses requires paradigm shifts which drive into a famous speech by Sherlock Holmes: “How often have I said to you that when you have eliminated the impossible, what remains, however improbable, must be the truth?” [63].

**Table 1:** Proposed clinical and laboratory criteria for rare and orphan diseases screening

**Clinical red flags for rare and orphan diseases**	
1.	Intermittent or persistent episodes of inflammation unexplained by infections or neoplasia
2.	Severe, persistent or unusual recurrent infections
3.	Multiple and/or severe autoimmunity at any age
4.	Family history of inherited conditions
5.	Multiorgan infiltrates in the absence of infection or neoplasia
6.	Joint hypermobility with or without evidence of vascular lesions such as aneurysm, dissection, or ischemia
7.	Organic signs of storage disease, including nephrotic syndrome, heart failure, hepatosplenomegaly, or peripheral neuropathy, in the absence of autoimmunity, infection, neoplasia, or drug reaction
8.	Unusual signs of vasculitis, cutaneous ischemia, or multiorgan symptoms unexplained by systemic vasculitides and with exclusion of secondary causes
9.	Atypical skin, neurological, or joint symptoms with epidemiological history of *Rickettsia* or *Borrelia* exposure
**Laboratory red flags for rare and orphan diseases**	
1.	Persistent or recurrent episodes of elevated inflammatory markers, such as ESR, CRP, or SAA, in the absence of neoplasia or infection
2.	Peripheral hypereosinophilia and/or eosinophilic tissue infiltration in the absence of neoplasia or adverse drug reaction
3.	Persistent complement reduction, hypogammaglobulinemia, deficient vaccine response, altered lymphocyte immunophenotyping in the absence of infection, neoplasia, or autoimmune disease activity
4.	Granulomatous or histiocytic tissue lesion in the absence of infection or neoplasia
5.	Tissue amyloid or lysosomal deposit in anatomopathological analyses
6.	Monostotic or polyostotic bone lesions documented by imaging or histopathology, including sclerotic, lytic, or mixed lesions in the absence of infection or neoplasia

*CRP* c-reactive protein, *ESR* erythrocyte sedimentation rate, *SAA* serum amyloid A

**Table 2:** Autoinflammatory diseases

Disease	Gene	Inheritance	Clinical features	Laboratory tests	Treatment
FMF and PAAND	*MEFV*	AR and AD	Fever, serositis, pleural effusion peritonitis, arthritis, erysipelas-like erythema (rash)	Increased IM during disease flares. In severe cases, renal biopsy may show amyloid deposits; Molecular analysis^#^	Colch, CS, IL-1i, TNFi, IL-6i
CAPS (NOMID/CINCA, MWS, FCAS)	*NLRP3*	AD	Fever, urticarial rash, aseptic meningitis, hearing loss, conjunctivitis, optical nerve atrophy, abnormal endochondral bone growth	Increased IM during disease flares. In severe cases, renal biopsy may show amyloid deposits; Molecular analysis^#^	CS, IL-1i
MKD	*MVK*	AR	Fever, abdominal pain, lymphadenopathy, rash, oral and genital ulcers, hepatosplenomegaly	Increased IM and IgD serum levels during disease flares. Elevated levels of urinary mevalonic acid; Molecular analysis^#^	CS, IL-1i, TNFi, I IL-6i, Colch
TRAPS	TNFRSF1A	AD	Fever, abdominal pain, migratory rash with fasciitis, periorbital rash, serositis, conjunctivitis	Increased IM during disease flares; Molecular analysis^#^	CS, TNFi, IL-1i, IL-6i, Colch
PAPA, hyperzincemia and hyper-calprotecnemia syndrome	PSTPIP1	AD (variable penetrance)	Destructive arthritis, neutrophilic dermatoses (especially pyoderma gangrenosum), inflammatory skinrash, myositis	Increased IM during disease flares; Molecular analysis^#^	CS, TNFi, IL-1i, IL-6i
VEXAS syndrome	UBA1	Somatic mutation	Fever, rash (neutrophilic dermatoses), interstitial lung disease, vasculitis, arthralgia, arthritis, polychondritis, thrombosis, serositis, myocarditis, hepatosplenomegaly	Increased IM during disease flares. Macrocytic anemia, thrombocytopenia, bone marrow vacuolization in myeloid and erythroid precursor cells; Molecular analysis^#^	CS, azacytidine, HSCT, IL-6i
Yao syndrome	*NOD2 IV28 + 158 or R702W*	AD (variable penetrance)	Fever, rash, uveitis, abdominal pain, diarrhea, arthritis, arthralgia, sicca-like features	Increased IM during disease flares. Spongiotic dermatitis on skin biopsy; Molecular analysis^#^	CS, TNFi, IL-1i
PFAPA	-----*	-----*	Fever, aphthosis, stomatitis, pharyngitis, and cervical adenitis, arthralgia, and myalgia	Increased IM during disease flares	CS, cimetidine, Colch, tonsillectomy, IL-1i
SAPHO syndrome	-----*	-----*	Synovitis, acne, palmoplantar pustulosis, sternocostoclavicular hyperostosis, osteitis, arthritis, uveitis, and conjunctivitis	Increased IM during disease flares	NSAID, CS, MTX, SSZ, bisphosphonate, TNFi
CRMO	-----*	-----*	Bone inflammation including localized skin redness (rare), warmth and/or swelling, and pain, palmoplantar pustulosis, acne and sacroiliitis	Increased IM during disease flares	NSAID, CS, MTX, SSZ, bisphosphonate, TNFi
Adult-onset Still’s disease	-----*	-----*	Fever, salmon-pink rash, arthritis, sore throat, pharyngitis, lymphadenopathy, serositis, hepatosplenomegaly, myocarditis	Increased IM during disease flares. Leukocytosis with neutrophilia; Increase in ferritin levels, positive biomarkers for MAS in severe cases; RF and ANA negative	CS, NSAID, MTX, CsA, IL-1i, TNFi, IL-6i
Schnitzler syndrome	-----*	-----*	Fever, chronic urticarial rash, arthralgia, arthritis, lymphadenopathy, bone pain (periostitis), hepatosplenomegaly	Increased IM during disease flares. Increased IgM or IgG immunoglobulin levels, neutrophilia, neutrophilic dermal infiltrate on skin biopsy	CS, IL-1i, IL-6i

*AD* autosomal dominant, AR autosomal recessive, *CAPS* cryopyrin-associated autoinflammatory syndromes, *Colch* colchicine, *CRMO* chronic recurrent multifocal osteomyelitis, *CS* corticosteroids, *CsA* cyclosporine, *FCAS* familial cold autoinflammatory syndrome, *FMF* familial mediterranean fever, *HSCT* hematopoietic stem cell transplantation, *IL-1* interleukin-1, *IL-1i* interleukin-1 inhibitors, *IL-6* interleukin-6, *IL-6i* IL-6 inhibitors, *IgD* immunoglobulin D, *IgM* immunoglobulin M, *IM* inflammatory markers (erythrocyte sedimentation rate, c-reactive protein and serum amyloid A), *MEFV* mediterranean fever gene, *MKD* mevalonate kinase deficiency, *MVK* mevalonate kinase gene, *NLRP3* NLR family pyrin domain containing 3, *NOD2* Nucleotide Binding Oligomerization Domain Containing 2, *NOMID/CINCA* neonatal-onset multisystem inflammatory disease/chronic infantile neurological cutaneous and articular, *NSAID* non-steroidal anti-inflammatory drugs, *MAS* macrophagic activation syndrome, *MTX* methotrexate, *MWS* Muckle–Wells syndrome, *PAAND* pyrin-associated autoinflammation with neutrophilic dermatosis, *PFAPA* periodic fever, aphthous stomatitis, pharyngitis and cervical adenitis, *SAA* serum amyloid A, *SAPHO syndrome* synovitis, acne, pustulosis, hyperostosis, and osteitis syndrome, *SSZ* sulfasalazine, *TNF* tumor necrosis factor, *TNFi* TNF inhibitors, *TNFRSF1A* tumor necrosis factor receptor superfamily, member 1A, *TRAPS* tumor necrosis factor receptor-associated periodic syndrome, *UBA1* ubiquitin like modifier activating enzyme 1, *VEXAS syndrome* vacuoles, E1 enzyme, X-linked, autoinflammatory, somatic syndrome

^#^Molecular analysis (sanger, next generation sequencing, whole exome sequencing)

----*no single gene or inheritance has been identified

**Table 3:** Other primary immune regulatory disorders (PIRD)

Disease	Gene	Inheritance	Clinical features	Laboratory tests	Treatment
CVID	*TNFRSF13; PIK3CD GOF*; PIK3R1; *PTEN; among others*	AR and AD	Severe recurrent infections, bronchiectasis, interstitial lung disease (GLILD), autoimmune manifestations (ITP, AIHA, rheumatoid arthritis, SLE)	Marked decrease of IgG and a marked decrease in at least one of the IgM or IgA, absent isohemagglutinins and/or poor response to vaccines; Molecular analysis^#^	Ig, antibiotic prophylaxis, immunosuppression in case of autoimmune features
ALPS¶	*TNFRSF6;* *TNFSF6;* *CASP 8; CASP 10; FADD*	AR and AD	Splenomegaly, lymphadenopathy, autoimmune cytopenia, increased lymphoma risk, bacterial and viral infections	IgG and IgA normal or increased, elevated serum FasL, IL-10, vitamin B12, increased TCR + CD4-CD8 double negative T cells; Molecular analysis^#^	mTORi, mycophenolate, rituximab, antibiotic prophylaxis in some cases
CTLA4 deficiency	*CTLA4*	AD (variable penetrance)	Autoimmune cytopenia, enteropathy, interstitial lung disease with lymphocytic infiltration, splenomegaly, lymphadenopathy recurrent infections, psoriasis, vitiligo	Cytopenia, Impaired function of Tregs, hypogammaglobulinemia, lymphocytic or granulomatous organ infiltration on biopsy; Molecular analysis^#^	Abatacept, Ig, antibiotic prophylaxis, HSCT
DADA2	*ADA2*	AR	Systemic vasculitis (polyarteritis nodosa), early-onset recurrent ischemic stroke and fever, livedo racemosus, recurrent infections, arthralgia, arthritis	Increased IM during disease flares, hypogammaglobulinemia, cytopenia in bone marrow failure (Diamond-Blackfan features), ANA positivity; Molecular analysis^#^	CS, TNFi, Ig, HSCT
HA20	*TNFAIP3*	AD	Oral and genital ulcers, GI manifestations, fever, arthritis, recurrent infections, uveitis, polyautoimmunity	Increased IM during disease flares, neutrophilia, pathergy positivity, positive ANA; Molecular analysis^#^	CS, TNFi, colchicine, cyclosporine, azathioprine
SAVI	*STING*	AD and AR GOF	Perniotic rash, small vessel vasculitis, severe interstitial lung disease, alveolar hemorrhage, arthritis, cerebral calcifications, nail clubbing	Increased IM during disease flares, lymphopenia, increased interferon signature and serum IgG, positive ANA, RF and ANCA; Molecular analysis^#^	CS, JAKi and IFNi, antifibrotic drugs, lung transplantation
COPA syndrome	*COPA*	AD	Arthritis, severe interstitial lung disease, alveolar hemorrhage, glomerulonephritis, autoimmune features	Increased IM during disease flares, increased interferon signature, positive ANA, RF and ANCA; Molecular analysis^#^	CS, JAKi and IFNi, antifibrotic drugs, lung transplantation
PLAID/ APLAID	*PLCG2*	AD GOF	Cold urticaria, atopic skin lesions granulomatous dermatitis, severe recurrent infections, autoimmune features, recurrent swelling of palms and feet	Increased IM during disease flares, hypogammaglobulinemia; Molecular analysis^#^	Antihistamines, CS, Ig, antibiotic prophylaxis
Type 1 interpheronopaties	*TREX1;* *DNASE2;* *SAMHD1;* *RNASEH2A.* *IFIH1.* among others	AD GOF AR LOF	Fever, encephalopathy, cerebral calcification, seizures, hydrocephalus, aortic and valvular calcifications, acro-osteolysis, dental abnormalities, joint contractures, cutaneous ulcers and perniotic lesions	Increased IM during disease flares, increased interferon signature, positive ANA, RF and ANCA; Molecular analysis^#^	CS, JAKi and IFNi
Proteasomopathies	*PSMB8.* *PSMB10;* *POMP;* *among others*	AR and AD (dominant negative effect). Digenic inheritance.	Fever, joint contractures, myositis, hepatosplenomegaly, lipodystrophy, cerebral calcifications and perniotic lesions	Increased IM during disease flares, increased interferon signature, positive ANA, hypergammaglobulinemia. Molecular analysis^#^	CS, JAKi and IFNi

*AD* autosomal dominant, *ADA2* adenosine deaminase 2, *AIHA* autoimmune hemolytic anemia, *ALPS¶* autoimmune lymphoproliferative syndrome, *ANA* antinuclear antibodies, *APLAID* autoinflammation, antibody deficiency, and immune dysregulation, *AR* autosomal recessive, *CASP 8* caspase 8, apoptosis-related cysteine protease, *CASP 10* caspase 10, apoptosis-related cysteine protease, *COPA* coatomer subunit alpha, *CS* corticosteroids, *CTLA4* cytotoxic T-lymphocyte associated protein 4, *CVID* common variable immunodeficiency, *CGD* chronic granulomatous disease, *CRP* c-reactive protein, *DADA2* deficiency of adenosine deaminase 2, *DNASE2* deoxyribonuclease 2, lysosomal, *ESR* erythrocyte sedimentation rate, *FADD* fas-associating protein with death domain, *FASL* Fas ligand, *GLILD* granulomatous and lymphocytic interstitial lung diseases, *GI* gastrointestinal, *GOF* gain-of-function, *HA20* haploinsufficiency A20, *HSCT* hematopoietic stem cell transplantation, *IFIH1* interferon induced with helicase C domain 1, *Ig* immunoglobulin, *IgA* immunoglobulin A, *IgG* immunoglobulin G, *IgM* immunoglobulin M, *IFN* interferons, *IFNi* IFN inhibitors, *IM* inflammatory markers (erythrocyte sedimentation rate, c-reactive protein and serum amyloid A), *ITP* Immune thrombocytopenia, *JAK* janus kinase, *JAKi* JAK inhibitors, *MTOR* mammalian target of rapamycin, *mTORi* mTOR inhibitors, *PIK3CD GOF* phosphoinositide 3-kinase delta isoform deficiency gain-of-function, *PLAID* PLCG2 associated antibody deficiency and immune dysregulation, *PLCG2* phospholipase C gamma 2, *POMP* proteasome maturation protein, *PSMB8* proteasome 20S subunit beta 8, *PSMB10* proteasome 20S subunit beta 10, *PTEN* phosphatase and tensin homologue gene, *SAA* serum amyloid A, *RNASEH2A* ribonuclease HI subunit A, *SAMHD1* SAM domain and HD domain-containing protein 1, *SAVI* STING-associated vasculopathy with onset in infancy, *SLE* systemic lupus erythematosus, *STING* stimulator of interferon genes protein, *TCR* T-cell receptor, *TMEM137* transmembrane protein 137, *TNF* tumor necrosis factor, *TNFAIP3* tumor necrosis factor, alpha induced protein 3, *TNFi* TNF inhibitors, *TNFSF6* tumor necrosis factor ligand superfamily member, *TNFRSF6* tumor necrosis factor receptor superfamily, member 6, *TNFRSF13B* TNF receptor superfamily member 13B, *TREG* regulatory T cells, *TREX1* three-prime repair exonuclease 1

^#^Molecular analysis (sanger, next generation sequencing, whole exome sequencing)

----*no single gene or inheritance has been identified

**Table 4:** Granulomatous diseases

Disease	Gene	Inheritance	Clinical features	Laboratory tests	Treatment
PGA	*NOD2*	AD	Uveitis, granulomatous synovitis, camptodactyly, ichthyosiform rash, fever	Increased IM during disease flares; Elevated ACE levels; Granulomatous infiltration on biopsy; Molecular analysis^#^	CS, TNFi, MTX
GLILD	Usually associated with CVID genes	AR and AD	Lymphocytic interstitial lung disease and follicular bronchitis/bronchiolitis, granulomatous lung disease, lymphadenopathy	Pulmonary function with restrictive pattern with a low DLCO value; Lymphocyte infiltrate and non-caseating granulomas on lung biopsy; Molecular analysis^#^	CS, RTX, AZA, HSCT
Chronic granulomatous disease	*CYBB; CYBA*	AR and XL	Severe and recurrent infections, McLeod phenotype, (IBD)-like colitis, cutaneous, hepatic and CNS abscess. Autoimmune features such as SLE	Increased IM during disease flares. Abnormal DHR neutrophil assay; Molecular analysis^#^; Granulomatous infiltration on biopsy	Antibiotic and antifungal prophylaxis, HSCT, gamma interferon, mesalazine CS, AZA
Sarcoidosis	----*	----*	Interstitial lung disease, arthritis, lymphadenopathy, erythema nodosum, Löfgren’s syndrome, lupus pernio, uveitis, neurological and cardiac involvement, uveoparotid fever (Heerfordt’s syndrome), sarcoid dactylitis with bone lesions (Perthes Jungling syndrome)	Negative tuberculin test or negative IGRA; Elevated serum ACE or serum lysozyme, lymphopenia, increased 1,25-dihydroxyvitamin D, hypercalciuria; Elevated CD4/CD8 ratio (> 3.5) in BLF; Granulomatous infiltration on biopsy	CS, MTX, AZA, leflunomide, TNFi
Drug-induced sarcoidosis-like reactions (DISRs)	----*	----*	Systemic granulomatous lesions indistinguishable from sarcoidosis induced by immune check point inhibitors, TNF-inhibitors, antiretroviral or interferon therapies	Negative tuberculin test or negative IGRA, elevated serum ACE or serum lysozyme, lymphopenia, increased 1,25-dihydroxyvitamin D, hypercalciuria; Elevated CD4/CD8 ratio (>3.5) in BAL; Granulomatous infiltration on biopsy	Withdrawal of the offending agent, CS, switch to another TNFi
Necrotizing sarcoid granulomatosis	----*	----*	Persistent fever, night sweats, weight loss, fatigue, interstitial lung disease, peripheral lymphadenopathy, arthralgia, skin lesions	Increased IM during disease flares, necrosis (coagulative or caseous) and vasculitis with granulomas on lung biopsy, negative mycobacteria culture, negative ANCA	CS, cyclophosphamide, AZA
Lymphomatoid granulomatosis	----*	----*	Multiple bilateral pulmonary nodules with evidence of central necrosis and/or cavitation. Scattered subcutaneous or dermal nodules, CNS, lung, and kidneys involvement	Atypical EBV-positive B cells admixed with a prominent background of mononuclear cells comprised of T cells, plasma cells, and histiocytes on biopsy	CS, RTX, IFN-α, chemotherapy, HSCT
GLUS syndrome¶	----*	----*	Prolonged fever, liver, bone marrow, spleen, and lymph nodes manifestations with a benign and recurrent course	Normal ACE, normal calcium levels; Predominantly B-cells in granuloma lesions on biopsy	CS

*ACE* angiotensin-converting enzyme, *AD* autosomal dominant, *ANA* antinuclear antibodies, *AR* autosomal recessive, *ANCA* antineutrophil cytoplasmic antibodies, *AZA* azathioprine, *BCG* bacillus Calmette–Guérin, *BAL* bronchoalveolar lavage, *CNS* central nervous system, *CRP* c-reactive protein, *CS* corticosteroids, *CYBA* cytochrome B-245 light chain, *CYBB* cytochrome B-245 beta chain, *DHR* dihydrorhodamine, *DLCO* diffusing capacity of lung for carbon monoxide, *EBV* Epstein-Barr virus, *ESR* erythrocyte sedimentation rate, *GLILD* granulomatous and lymphocytic interstitial lung diseases, *GLUS syndrome¶* granulomatous lesions of unknown significance syndrome, *HSCT* hematopoietic stem cell transplantation, *IBD-like colitis* inflammatory bowel disease-like colitis, *IGRA* interferon-gamma releasing assays, *IFN-α* interferon alpha therapy, *IM* inflammatory markers (erythrocyte sedimentation rate, c-reactive protein and serum amyloid A), *MTX* methotrexate, *NOD2* Nucleotide Binding Oligomerization Domain Containing 2, *PGA* pediatric granulomatous arthritis, *RTX* rituximab, *SAA* serum amyloid A, *SLE* systemic lupus erythematosus, *TNF* tumor necrosis factor, *TNFi* TNF-inhibitors, *XL* X-linked inheritance

^#^Molecular analysis (sanger, next generation sequencing, whole exome sequencing)

----*no single gene or inheritance has been identified

**Table 5:** Histiocytoses disorders

Disease	Gene	Inheritance	Clinical features	Laboratory tests	Treatment
HLH	*PRF1;* *UNC13D;* *STX11;* *STXBP2.*	AR	Fever, hepatosplenomegaly, lymphadenopathy, cytopenia, serositis, hepatic and CNS involvement	Increased levels of ferritin, triglycerides, d-dimer; Increased CRP, SAA, low ESR during disease flares; Cytopenia, low levels of fibrinogen, inactivity of natural killer cells, increased sIL2R; Presence of hemophagocytosis on myelogram or tissue biopsy	CS, IL-1i, Ig, etoposide, HSCT; Control of infectious agents
Langerhans cell histiocytosis¶	*BRAF V600E;* *MAPK.*	Somatic mutation	Pruritic or ulcerative skin rashes, interstitial lung disease (cystic pattern), liver, spleen and/or bone marrow involvement	CD1a + Langerin + Langerhans cell Infiltration, CD1a + Langerin with + with BRAF V600E+ (50-60%) and eosinophils; Electron microscopy with Birbeck granules	Vinblastine + CS/ Cladribine + Cytarabine, Vemurafenib; Quit smoking
Erdheim-Chester disease	*BRAF V600E;* *MAPK.*	Somatic mutation	Diaphyseal osteolytic lesions, coated aorta, renal lesion (hairy kidney), proptosis, cardiac and nervous system involvement	Touton cells on biopsy, histiocytes stained with CD68 + and CD1a- and S100- markers, BRAF V600E positive in tissue sample	Gamma interferon, Cyclophosphamide, Vemurafenib, Cladribine, Infliximab, Anakinra, Tocilizumab
Rosai-Dorfman disease	*MAPK;* *KRAS.*	Somatic mutation	Localized or disseminated painful lymph node enlargement, retroorbital or skin involvement., autoimmune or neoplastic manifestations may be associated	Sinusoidal expansion in the lymph nodes; Emperipolesis or storiform fibrosis may be present in the tissue; Histiocytes CD68+, CD1a -, S100+; IgG4 + in some cases	Sporadic remission in 50% of cases; Unifocal: Surgical excision; Multifocal: CS, Sirolimus, Chemotherapy, MAPK inhibitor
Kikuchi-Fujimoto disease	----*	----*	Fever, painful lymphadenopathy usually cervical, hepatosplenomegaly, arthralgia. may be associated with autoimmune diseases (SLE)	Increased IM during disease flares, leukopenia, atypical lymphocytes in peripheral blood. Clusters of small lymphocytes, histiocytes CD68+, immunoblasts and plasma cells in the absence of neutrophils with necrosis; Abundant karyorrhectic	Sporadic remission for self-limited disease, CS or surgical excision for severe clinical features
Multicentric reticulohistiocytosis	----*	----*	Skin papules and nodules on face, periungual nodules and papules (coral bead appearance), fever, xanthelasma, destructive arthritis (proximal interphalangeal DIP predominance); Autoimmune and neoplastic manifestations diseases may be associated	Increased IM during disease flares, hyperlipidemia and hypergammaglobulinemia; Positive RF, ANA, or CCP is rarely observed. Histiocytic multinucleated CD68+, CD1a-, S100-	CS, methotrexate, bisphosphonates, cyclophosphamide, TNF-inhibitors, Chemotherapy in cases with malignancy
Macrophage activation syndrome	----*	----*	Fever, hepatosplenomegaly, cytopenia, lymphadenopathy, hepatic and nervous system involvement	Increased levels of ferritin, triglycerides, d-dimer, cytopenia, low levels of fibrinogen, low/absent NK cell activity, increased sIL2R; Presence of hemophagocytosis on myelogram or tissue biopsy	CS, Ig, IL-1i, tocilizumab, cyclosporine, etoposide, control of infectious agents

*AR* autosomal recessive, *BRAF* B-Raf proto-oncogene, serine/threonine kinase V600E, *CCP* anti-cyclic citrullinated peptide, *CD1a+* cluster of differentiation 1a+, *CD68* cluster of differentiation 68, *CRP* c-reactive protein, *CS* corticosteroids, *DIP* distal interphalangeal, *ESR* erythrocyte sedimentation rate, *HLH* hemophagocytic lymphohistiocytosis, *HSCT* hematopoietic stem cell transplantation, *Ig* immunoglobulin, *IgG4* immunoglobulin 4, *IFNγ*. interferon-gamma, *IL-1* Interleukin-1, *IL-1i* IL-1 inhibitors, *IL-2 soluble* interleukin-2 soluble, *IM* inflammatory markers (erythrocyte sedimentation rate, c-reactive protein and serum amyloid A), *KRAS* kirsten rat sarcoma viral oncogene homolog, *Langerhans cell histiocytosis¶* Hand-Schuller-Christian or Letterer-Siwe disease or histiocytosis X, *MAPK* mitogen-activated protein kinase 1, *PRF1* perforin 1, *NK* Natural killer cells, *RF* rheumatoid factor, *SAA* serum amyloid A, *SLE* systemic lupus erythematosus, *Soluble IL-2* soluble interleukin-2, *sIL2R* soluble interleukin-2 receptor, *STX 11* syntaxin-11, *STXBP2* syntaxin binding protein 2, *TNF-inhibitors* tumor necrosis factor-alpha inhibitors, *UNC13D* Unc-13 Homolog D

----*no single gene or inheritance has been identified

**Table 6:** Primary connective tissue diseases

Disease	Gene	Inheritance	Clinical features	Laboratory tests	Treatment
Ehlers-Danlos syndrome	*COL1A1;* *COL1A2;* *COL3A1*,* COL5A1;* *COL5A2;* *TNXB*,* and others*	AD and AR	Skin hyperextensibility with atrophic scarring, generalized joint hypermobility, exercise intolerance, muscle weakness, arterial rupture at young age, spontaneous sigmoid colon, or uterine rupture; MCAS and POTS may be associated	Molecular analysis^#^; Increased serum tryptase or urinary N-MH, PG metabolites, LTE4, ≥ 20 MCs per HPF in extracutaneous tissue biopsy in case of MCAS	Angiotensin receptor blocker and/or beta-blocker for aneurysm, pain relief drugs, physiotherapy, and rehabilitation therapy; Surgical intervention (aneurysm, dissection, organ rupture); Antihistamines, leukotriene antagonists, 5-lipoxygenese inhibitors, and PG blockers in case of MCAS
Marfan syndrome	*FBN1*	AD	Wrist and thumb sign, arachnodactyly, generalized joint hypermobility, anterior chest deformity (pectus excavatum and/or carinatum), pneumothorax, myopia, ectopia lentis, aortic root dilatation	Molecular analysis^#^. Aortic dilation (Z-score ≥ 2 SD) on echocardiography	Angiotensin receptor blocker and/or b-blocker for aneurysm, pain relief drugs, physiotherapy, and rehabilitation therapy; Surgical intervention (aneurysm, dissection)
Loeys-Dietz syndrome	*TGFBR1;* *TGFBR2;* *TGFB2;* *TGFB3;* *SMAD3;* *SMAD2.*	AD	Generalized joint hypermobility, chest deformity, scoliosis, bifid (split or forked) uvula, cleft palate, hypertelorism; Aortic root aneurysms, arterial tortuosity, dissections, mitral valve prolapse	Molecular analysis^#^	Angiotensin receptor blocker and/or b-blocker for aneurysm, pain relief drugs, physiotherapy, and rehabilitation therapy; Surgical intervention (aneurysm, dissection)

*AD* autosomal dominant, *AR* autosomal recessive, *COL1A1* Collagen Type I Alpha 1 Chain, *COL1A2* Collagen Type I Alpha 2 Chain, *COL3A1* Collagen Type III Alpha 1 Chain, *COL5A1* Collagen Type V Alpha I Chain, *COL5A2* Collagen Type V Alpha II Chain, *Echo* echocardiogram, *FBN1* fibrillin 1, *HPF* high-power field, *LTE4* leukotriene E4, *POTS* postural orthostatic tachycardia syndrome, *MC* mast cell, *MCAS* mast cell activation syndrome, *N-MH* N-methylhistamine, *PG* prostaglandin, *SD* standard deviation, *SMAD2* SMAD family member 2, *SMAD3* SMAD family member 3, *TGFB2* transforming growth factor beta 2, *TGFB3* transforming growth factor beta 3, *TGFBR1* transforming growth factor beta receptor 1, *TGFBR2* transforming growth factor beta receptor 2, *TNXB* Tenascin-X

^#^Molecular analysis (sanger, next generation sequencing, whole exome sequencing)

**Table 7:** Storage disease

Disease	Gene	Inheritance	Clinical features	Laboratory tests	Treatment
Fabry disease	*GLA*	XL	Acroparesthesia, peripheral neuropathy, angiokeratoma, proteinuria, kidney failure, myocardial hypertrophy, arrhythmia, cornea verticillata, febrile crisis, strokes, and TIA	Absent or reduced GLA activity. Increased serum lyso-Gb3; Molecular analysis^#^; Zebra bodies (electron microscopy) with Gb3 deposits in tissue biopsy (kidney or heart)	ERT (agalsidasealfa, agalsidase-beta, pegunigalsidase alfa), migalastat for amenable variants, pain relief drugs, ACEI or ARB to target albuminuria
Deficiency of glucocerebrosidase	*GBA*	AR	Hepatosplenomegaly, anemia, thrombocytopenia, bone pain, osteopenia, fractures, growth retardation, neurological involvement, neonatal death	Anemia and/or thrombocytopenia; Presence of Gaucher cells in bone marrow aspiration; Decreased GCase activity; Molecular analysis^#^	ERT (imiglucerase, velaglucerase alfa, and taliglucerase alfa), SRT (miglustat, eliglustat). Pain relief drugs
Mucopolysaccharidosis I¶	*IDUA*	AR	Dysmorphic features, hernias, thoracolumbar kyphosis, and hepatosplenomegaly, hearing loss, cognitive impairment, cardiac and respiratory manifestation	Decreased IDUA enzyme assay; Increased urine GAG; Molecular analysis^#^	ERT (laronidase), HSCT
Acid ceramidase deficiency	*ASAH1*	AR	Subcutaneous nodules, joint pain, and voice hoarseness	Decreased ACDase activity; Molecular analysis^#^; Farber bodies in tissue biopsy	Pain relief drugs
ATTR amyloidosis (ATTRwt and ATTRv)	*TTR* (ATTRv)	AD (ATTRv)	Peripheral polyneuropathy, dysautonomia, congestive heart failure, untreatable arrhythmia; Carpal tunnel syndrome, lumbar spinal stenosis, trigger finger, tendon rupture, arthralgia	Increased BNP, NT-proBNP, Troponin-T; Positive 99mTc-PYP scan; Congo red positivity on tissue biopsy; Molecular analysis^#^	Silencers (patisiran and inotersen), stabilizers (tafamidis and diflunisal); Pain relief drugs, antiarrhythmic agents, ACEI or ARB
AA amyloidosis	----*	----*	Proteinuria, nephrotic syndrome, malabsorption with chronic diarrhea, acute obstruction or bleeding, hepatomegaly, cardiac and neuropathic involvement are both extremely rare	Increased IM during disease flares, Congo red positivity on tissue biopsy; Positive 123 labeled SAP scintigraphy	Control of the underlying disease; Colchicine prophylaxis in FMF

*AA amyloidosis *inflammatory or secondary amyloidosis, *ACDase *acid ceramidase, *ACEI *angiotensin-converting-enzyme inhibitors, *AD *autosomal dominant, *AR *autosomal recessive, *ARB *angiotensin receptor blockers, *ATTR amyloidosis *transthyretin amyloidosis, *ASAH1 *N-Acylsphingosine Amidohydrolase 1, *BNP *brain natriuretic peptide, *CRP* c-reactive protein, *ERT* enzyme replacement therapy, *ESR* erythrocyte sedimentation rate, *FMF* familial mediterranean fever, *GAA* glucosidase alpha acid, *GAG* glycosaminoglycans, *GBA* glucosylceramidase beta 1, *GCase* acid beta-glucosidase, *GLA* galactosidase alpha, *I123 laveled* Iodine-123 labeled, *IDUA* alpha-L-Iduronidase, *IM* inflammatory markers (erythrocyte sedimentation rate, c-reactive protein and serum amyloid A), *Lyso-Gb3* globotriaosylsphingosine, *Mucopolysaccharidosis I¶* mild (Scheie syndrome), intermediate (Hurler-Scheie syndrome), severe (Hurler syndrome), *NT-proBNP* N-terminal prohormone of brain natriuretic peptide, *SAA* serum amyloid A, *SAP scintigraphy* serum amyloid P scintigraphy, *SRT* substrate reduction therapy, *TIA* transient ischemic attack, TTR transthyretin, *XL* X-linked inheritance, *99mTc-PYP* (technetium-99 pyrophosphate scintigraph

^#^Molecular analysis (sanger, next generation sequencing, whole exome sequencing)

----*no single gene or inheritance has been identified

**Table 8:** Metabolic bone diseases

Disease	Gene	Inheritance	Clinical features	Laboratory tests	Treatment
Hypophosphatasia	*ALPL*	AR and AD	Infantile: polyhydramnios, low or absent skeletal mineralization; Childhood: premature tooth loss, bone pain, muscle weakness; Adults: multiple fractures (metatarsal stress fractures are common), periarticular calcifications (CPPD), bone pain; Dental: premature tooth loss, poor dental health	Low serum ALP levels; Increased serum PLP levels. Increased urinary PEA levels; Molecular analysis^#^	ERT (asfotase alfa), Adjust calcium and vitamin D, pain relief drugs; Rehabilitation, dental care
Fibrodysplasia ossificans progressiva	*ACVR1*	AD	Congenital bilateral hallux valgus; Early-onset heterotopic ossification, which may be spontaneous or precipitated by trauma; Painful, recurrent soft-tissue swellings	Molecular analysis^#^	Avoid intramuscular injections and arterial punctures; Fall prevention; Corticosteroids for flare-ups. Palovarotene
Osteogenesis imperfecta&	*COL1A1;* *COL1A2*,* and others*	AD and AR and XL	Blue sclera, multiple recurrent fractures in atypical locations, scoliosis, short stature, restricted mobility with respiratory complications, muscle weakness, hearing loss dentinogenesis imperfecta	Molecular analysis^#^	Bisphosphonates, denosumab, teriparatide, orthopedic surgery
Pachydermoperiostosis*	*HPGD;* *SLCO2A1.*	AR or AD	Digital clubbing, bone pain (periostosis), arthralgia, cutis verticis gyrate, seborrhea, hyperhidrosis	Molecular analysis^#^	NSAID, bisphosphonates
Osteopoikilosis	*LEMD3*	AD	Typically, asymptomatic; Localized sclerotic epi/metaphyseal lesions with “spotty” bones; Dermatofibrosis lenticularis (Buschke-Ollendorff syndrome)	Molecular analysis^#^	Supportive, bisphosphonates in rare cases for pain
Melorheostosis	*MAP2K1* *LEMD3*,* KRAS*,* SMAD3*	Somatic	Incidental radiographic findings such as cortical sclerotic lesions, “dripping candle wax” appearance; Myositis ossificans like appearance associated soft tissue involvement with joint contractures	Molecular analysis^#^	Supportive, pain modulation, bisphosphonates for pain, rehabilitation
Camurati–Engelmann disease¶	*TGFB1*	AD	Leg pain, muscle weakness, and easy fatigability; Symmetric thickening of long bone diaphysis with hyperostosis	Molecular analysis^#^	Corticosteroids may relieve the pain, losartan (controversial); Orthopedic surgery

*ACVR1* activin A receptor type 1, *AD* autosomal dominant, *ALP* alkaline phosphatase, *ALPL* alkaline phosphatase, biomineralization associated, *AR* autosomal recessive, *Camurati–Engelmann disease*¶ also known as progressive diaphyseal dysplasia, *COL1A1* collagen type I alpha 1 chain, *COL1A2* collagen type I alpha 2 chain, *CPPD* calcium pyrophosphate crystal deposition, *ERT* enzyme replacement therapy, *HPGD* 15-hydroxyprostaglandin dehydrogenase, *HSCT* hematopoietic stem cell transplantation, *LEMD3* LEM domain containing 3, *MAP2K1* mitogen-activated protein kinase 1, *NSAID* non-steroidal anti-inflammatory drugs, *Osteogenesis imperfecta&* also known as brittle bone disease, Pachydermoperiostosis* also known as primary hypertrophic osteoarthropathy, *PEA* phosphoethanolamine, *PLP* pyridoxal 5-phosphate or vitamin B6, *SLCO2A1* solute carrier organic anion transporter family, member 2A1, *TGFB1* transforming growth factor beta1

^#^Molecular analysis (sanger, next generation sequencing, whole exome sequencing)

**Table 9:** Rare vasculopathies

Disease	Gene	Inheritance	Clinical features	Laboratory tests	Treatment
PACNS	----*	----*	Headache, cognitive impairment, stroke or TIA, focal neurological deficits, seizure, Impaired level of consciousness, psychiatric or mood disorders	CSF analysis: lymphocytic pleocytosis and elevated protein levels; Neuroimaging: Multiple ischemic lesions, intraparenchymal or subarachnoid hemorrhage, vessel wall enhancement (black blood MRI); Brain biopsy: necrotizing, lymphocytic, or granulomatous (amyloid beta-related vasculitis may be associated)	Induction: CS, CYC; Remission: AZA or MMF
Cogan syndrome	----*	----*	Sudden onset tinnitus and vertigo, hearing loss (Meniere-like features); Nystagmus, non-syphilitic interstitial keratitis, uveitis, episcleritis, scleritis, optic neuritis, aortitis (Takayasu-like features)	Increased IM during disease flares; Currently, no specific serological biomarker is available	CS, TNFi, CYC, MTX, CsA, MMF, and AZA
Susac syndrome	----*	----*	Headache (migraine pattern), subacute encephalopathy, severe confusional state with behavioral disturbances, psychiatric manifestations, multiple BRAO (gass plaques), sensorineural hearing loss	Neuroimaging: snowball lesion in corpus callosum; CSF: lymphocytic pleocytosis, increase levels of protein, oligoclonal bands are rare; Retinal fluorescein angiography with multiple BRAO; Audiogram: hearing loss predominating in the low to midtone range frequencies	CS, CYC, AZA, MMF, RTX, anti-platelet agents
Vogt-Koyanagi-Harada disease	----*	----*	Fever, malaise, nausea, bilateral granulomatous panuveitis, iris lesion (Sugiura sign), eyelash whitening (poliosis), vitiligo, hearing loss, meningismus, tinnitus	OCT with exudative retinal detachments; Indocyanine green angiography or fluorescein angiogram with retinal depigmentation (Dalen Fuchs nodules and sunset glow fundus)	CS, AZA, CYC, MTX, CsA and MMF
IgG4-RD	----*	----*	Major salivary and lacrimal gland enlargement, orbital disease, hypophysitis, autoimmune pancreatitis, tubulointerstitial nephritis, thyroiditis, mediastinal fibrosis, hypertrophic pachymeningitis and retroperitoneal fibrosis	Mild peripheral eosinophilia, increased IgG4 and IgE blood levels; Decreased C3 and C4 complement in disease flare; Biopsy: Dense lymphoplasmacytic infiltrate, elevated numbers of IgG4 positive cell, IgG4 to IgG ratio > 40%, storiform fibrosis, obliterative phlebitis	CS, RTX, AZA, MMF, CYC
Relapsing polychondritis	----*	----*	Bilateral auricular chondritis, nasal chondritis, respiratory tract chondritis, non-erosive seronegative inflammatory polyarthritis, uveitis, conjunctivitis, necrotizing scleritis, hearing loss. MAGIC syndrome, VEXAS or MDS may be associated	Increased IM during disease flares; Dynamic CT chest expiratory airway abnormalities; Bronchoscopy: potential airway collapse or stenosis	CS, NSAID, dapsone, AZA, TNFi, RTX and CYC
Livedoid vasculopathy	----*	----*	Chronic, recurrent thrombo-occlusive disease of the veins, livedo racemosa and Milian white atrophy; Primary thrombophilia or APS may be associated	Primary thrombophilia lab and anti-phospholipid antibodies should be tested; Biopsy: intraluminal thrombosis, endothelial proliferation, subintimal hyaline degeneration	Aspirin, anticoagulants. HOT, CS, AZA, HCQ, CsA and CYC
Kohlmeier-Degos disease¶	----*	----*	Atrophic skin lesions, porcelain-white papules with peripheral telangiectasias, mesenteric ischemia and strokes	Biopsy: skin atrophy with thrombotic vasculopathy, rich mucin deposit and presence of C5-9 complement	CS, prostaglandin agonists, anticoagulation and eculizumab
Juvenile temporal arteritis	----*	----*	Usually affects individuals younger than 50 with asthenia, headache or visual blur and a lump in the temporal region	Peripheral blood eosinophilia. Normal IM; Temporal artery biopsy: panarteritis with a prominent eosinophilic infiltrate, granulomatous lesions or giant cells are rare	Spontaneously remission; Relapsing: CS, NSAID, colchicine and complete excision

*APS* antiphospholipid syndrome, *AZA* azathioprine, *BRAO* branch retinal artery occlusion, *CS* corticosteroids, *CsA* cyclosporine, *CRP* c-reactive protein, *CSF* cerebrospinal fluid, *CYC* cyclophosphamide, *ESR* erythrocyte sedimentation rate, *HOT* hyperbaric oxygen therapy, *HCQ* hydroxychloroquine, *IgE* immunoglobulin E, *IgG* immunoglobulin G, *IgG4* immunoglobulin G4, *IgG4 RD* immunoglobulin G4-related disease, *IM* inflammatory markers (erythrocyte sedimentation rate, c-reactive protein and serum amyloid A), *Kohlmeier-Degos disease¶* also known as malignant atrophic papulosis, *NSAID* non-steroidal anti-inflammatory drugs, *MAGIC syndrome* mouth and genital ulcers with inflamed cartilage syndrome, *MDS* myelodysplastic syndromes, *MMF* mycophenolate, *MRI* magnetic resonance imaging, *MTX* methotrexate, *OCT* optical coherence tomography, *PACNS* primary angiitis of the central nervous system, *RCVS* reversible cerebral vasoconstriction syndrome, *RTX* rituximab, *SAA* serum amyloid A, *TIA* transient ischemic attack, *TNFi* tumor necrosis factor inhibitors, *VEXAS* vacuoles in blood cells, the E1 enzyme, X-linked, autoinflammatory, and somatic

----*no single gene or inheritance has been identified

**Table 10:** Hypereosinophilic syndromes

Disease	Gene	Inheritance	Clinical features	Laboratory tests	Treatment
EGPA	----*	----*	Sinus polyps, asthma, migratory pulmonary infiltrates, necrotizing glomerulonephritis, peripheral neuropathy (mononeuritis multiplex), cutaneous vasculitis and myocarditis	Peripheral blood eosinophilia, increased IgE. ANCA-p positivity, MPO positivity, PR-3 positivity (rare); Kidney biopsy: pauci-immune glomerulonephritis; Skin or lung biopsy: non-caseating granulomas, perivascular eosinophil infiltrates	Induction: CS or avacopan with CYC or RTX, PLEXˆ not routinely recommended; Remission: RTX, MEP, AZA, MMF
Eosinophilic fasciitis&	----*	----*	Skin tightening, peau d’orange pattern, skin groove sign, prayer sign, muscle weakness; Benign or malignant hematologic abnormalities	Peripheral blood eosinophilia, hypergammaglobulinemia; Increased aldolase and CK levels; MRI: abnormal fascial signal intensity and enhancement; Deep skin biopsy: eosinophilic infiltrates with fasciitis	CS, MTX, CYC, AZA, cyclosporine and RTX
Eosinophilic myositis	----*	----*	Pain and calf swelling (other muscles can be affected), proximal weakness	Peripheral blood eosinophilia, increased aldolase and CK levels; Biopsy: Deep mononuclear cell infiltration (eosinophilic or not), with muscle fiber invasion and necrosis on muscle biopsy	Spontaneously remission; Relapsing: CS, NSAID
Kimura’s disease¶	----*	----*	Painless head and/or neck lymphadenopathy or salivary gland enlargement (more common in Asian population); Allergic skin disease and glomerulonephritis has been associated in few cases	Peripheral blood eosinophilia, increased IgE; Biopsy: nodular, diffuse, and mixed inflammatory infiltrate composed mainly of lymphocytes and eosinophils, lymphoid follicles are hyperplastic and contain prominent germination centers	CS, radiotherapy, surgical resection
ALHE	----*	----*	Dermal papules and nodules generally in head and neck region (more common in Asian population); Orbital region may be involved	Absent of peripheral blood eosinophilia, normal IgE serum levels; Biopsy: numerous enlarged endothelial cells in the lumen of an abnormal blood vessel; Presence of lymphocytes and eosinophils	Surgical resection; isotretinoin, laser, cryotherapy, imiquimod
PPP syndrome	----*	----*	Panniculitis, arthritis and/or bone necrosis, acute or chronic pancreatitis or pancreatic tumors; Fever, abdominal pain, vomiting and nausea	Peripheral blood eosinophilia Biopsy: lobular non-vasculitis panniculitis	CS, NSAID; Control the underlying pancreatic disease
RCNEV	----*	----*	Annular urticarial plaques, purpuric papules, angioedema, chronic relapsing process; Absence of any features of systemic disease	Peripheral blood eosinophilia; Biopsy: necrotizing vasculitis of dermal small vessels with prominent eosinophilic infiltration; Granulomas and leukocytoclastic features are typically absent	CS, NSAID, AZA, MTX and tacrolimus
Strongyloidiasis	----*	----*	Diarrhea, weight loss, epigastric pain, pneumonia, cutaneous periumbilical purpura, septic shock (hyperinfection syndrome)	Peripheral blood eosinophilia; Serologic detection of Strongyloides specific antibodies; Microscopic detection of larvae in stool with BM or APC culture or qPCR; Biopsy: microscopic detection of larvae in colonic tissue	Mild cases: ivermectin; Severe: ivermectin + albendazole
Loeffler syndrome	----*	----*	Dyspnea, cough, wheezing, pneumonia, more rarely myalgia, anorexia, hemoptysis and urticaria	Peripheral blood eosinophilia. BAL with increased eosinophil count; Serologic detection of Strongyloides specific antibodies; Microscopic detection of parasites larvae of A. lumbricoides or N. americanus or A. duodenale or S. stercoralis	Ivermectin or albendazole

*ALHE* angiolymphoid hyperplasia with eosinophilia, *ANCA-p* antineutrophil cytoplasmic antibodies- perinuclear pattern, *APC* agar plate culture, *AZA* azathioprine, *anti-PR3* anti-proteinase-3, *A. duodenale* Ancylostoma duodenale, *A. lumbricoides* Ascaris lumbricoides, *BAL* bronchoalveolar lavage, *BM* Baermann modification, *CS* corticosteroids, *CK* creatine kinase, *CYC* cyclophosphamide, *EGPA* also known as eosinophilic granulomatosis with polyangiitis or Churg Strauss syndrome, *Eosinophilic fasciitis&* also known as Shulman syndrome, *IgE* immunoglobulin E, *Kimura’s disease¶* eosinophilic hyperplastic lymphoid granuloma, *MEP* mepolizumab, *MMF* mycophenolate, *MPO* anti-myeloperoxidase antibody, *MTX* methotrexate, *NSAID* non-steroidal anti-inflammatory drugs, *N. americanos* Necator americanus, *PLEXˆ* also known as plasmapheresis or apheresis or plasma exchange, *PPP syndrome* pancreatitis, panniculitis and polyarthritis syndrome, *PR3* anti-proteinase-3 antibody, *qPCR* quantitative polymerase chain reaction, *RCNEV* recurrent cutaneous necrotizing eosinophilic vasculitis, *RTX* rituximab, *S. stercoralis* Strongyloides stercoralis

----*no single gene or inheritance has been identified

**Table 11:** Adjuvant-induced rheumatic diseases

Disease	Etiology	Gene	Clinical features	Laboratory tests	Treatment
ASIA syndrome - Siliconosis	Silicone (breast implant)	HLA-DRB1; and HLA-DQB1	Weakness, myalgia, or myositis, arthritis or arthralgia, chronic fatigue, malaise, or sleep disturbances, neurological manifestations related to demyelination, cognitive deficits, fever, dry mouth, or other sicca syndrome–like symptoms	Positivity for autoantibodies or antibodies targeting the suspected adjuvant; High-resolution histocompatibility HLA-DR and HLA-DQ	Removal of the adjuvant leads to a full or at least partial recovery
ASIA syndrome - Post-vaccination/ MMF	Aluminum	HLA-DRB1; and HLA-DQB1	Systemic: weakness, myalgia, or myositis, arthritis or arthralgia, chronic fatigue, malaise, or sleep disturbances, neurological manifestations related to demyelination, cognitive deficits, fever, dry mouth, or other sicca syndrome–like symptoms; Local: active lesion at the site of inoculation	Positivity for autoantibodies or antibodies targeting the suspected adjuvant; CK elevated levels. High-resolution histocompatibility HLA-DR and HLA-DQ; Muscle biopsy with PAS-positive, MHC-1-positive macrophages; Aluminum hydroxide engulfed by macrophages in EM	Avoid repeated exposure to triggering adjuvants
ASIA syndrome - Gulf War syndrome	Sarin gas? Pesticides? Vaccine?	HLA-DRB1; and HLA-DQB1	Fatigue, joint pain, headaches, rashes or skin problems, insomnia	Presence of antibodies to the adjuvant squalene correlated (lack of standardization); High-resolution histocompatibility HLA-DR and HLA-DQ	Removal of the adjuvant leads to a full or at least partial recovery
Eosinophilia-myalgia syndrome	L-tryptophan	----*	Severe muscle pain, cough or dyspnea, fever, rash, arthralgia, thickened skin, myocarditis, neuropathy	Peripheral blood eosinophilia; Histopathology with perimyositis with inflammatory cells predominantly mononuclear cells and eosinophils	Removal of the adjuvant leads to a full or at least partial recovery
Toxic oil syndrome	Aniline rapeseed oil	----*	Diffuse interstitial or alveolar interstitial infiltrates, pulmonary hypertension, incapacitating myalgia, cardiomyopathy, and skin rash	Peripheral blood eosinophilia; Imaging tests: diffuse interstitial or alveolar interstitial infiltrates, with or without pleural effusion	Removal of the adjuvant leads to a full or at least partial recovery
Nephrogenic systemic fibrosis	Gadolinium	----*	Severe skin thickening after gadolinium exposure with joint contracture	Skin biopsy: Fibrosis of the dermis with extension to the subcutaneous fat pads; Notably absent inflammatory infiltrate	Preventing gadolinium exposure in chronic kidney patients
Metallosis	Cobalt and chromium	----*	Vision and hearing impairment, fatigue, peripheral neuropathy, weight loss, cardiotoxicity, skin rash and hypothyroidism	Co and Cr increased blood levels; Increased thyrotropin. Imaging tests with joint metallosis	Removal of the Co and Cr prosthesis; Chelation therapy
Vinyl chloride monomer related diseases	VCM	----*	Acro-osteolysis, thickened skin, vascular alterations (Raynaud’s phenomenon), liver cirrhosis, hepatocellular carcinoma, arthralgia, and myalgia	Increased urinary TDGA levels	The removal of the adjuvant leads to a full or at least partial recovery
PPD induced arthritis	PPD	----*	Symmetrical polyarthritis after PPD exposure; Allergic contact dermatitis and/or angioedema	Increased IM during disease flares, positive patch test for PPD	The removal of the adjuvant leads to a full or at least partial recovery

*ASIA syndrome* also known as autoimmune/inflammatory syndrome induced by adjuvants syndrome or Shoenfeld’s syndrome, *CK* creatine kinase, *Co/Cr* cobalt and chromium, *CRP* c-reactive protein, *EM* electron microscope, *ESR* erythrocyte sedimentation rate, *HLA-DQB1* major histocompatibility complex, class II, DQ beta 1, *HLA-DRB1* major histocompatibility complex, class II, DR beta 1, *IM* inflammatory markers (erythrocyte sedimentation rate, c-reactive protein and serum amyloid A), *L-tryptophan* levorotatory form of tryptophan, *MHC1* major histocompatibility complex class 1, *MMF* macrophagic myofasciitis, *PAS* periodic acid Schiff, *PPD* para-phenylenediamine, *SAA* serum amyloid A, *TDGA* thiodiglycolic acid, *VCM* vinyl chloride monomer

----*no single gene or inheritance has been identified

**Table 12:** Rare infectious/parasitic diseases

Disease	Etiology	Transmission	Clinical features	Laboratory tests	Treatment
Whipple disease	*T. whipplei*	Exposure to contaminated soil	Weight loss, diarrhea, fever, lymphadenopathy, skin hyperpigmentation, arthritis, heart failure, CNS involvement (oculomasticatory myorhythmia or oculo-facio-skeletal myorhythmia)	PAS test from duodenal/jejunal biopsy tissue; Positive T. whipplei PCR in stools, CSF, or saliva	Ceftriaxone or Meropenem 2 weeks, co-trimoxazole or doxycycline with hydroxychloroquine for 1 year. Corticosteroids with IRIS present
Lyme disease	*B. burgdorferi*	Tick-borne (I. scapularis or I. pacificus) in USA, Europe, and Asia	Erythema migrans (bull- eye pattern), arthritis, myocarditis, peripheral nervous system, facial palsy, meningoencephalitis, chronic fatigue syndrome; Autoimmune and allergic diseases development	ELISA IgM and IgG for B. burgdorferi, western blot IgM and IgG for B. burgdorferi	Ceftriaxone intravenously for severe features. Doxycycline or amoxicillin for mild features
Baggio-Yoshinari syndrome¶	*B. burgdorferi sensu stricto spirochetes*	Tick-borne (A. cajennense or R. sanguineus or D. nitens) in Brazil	Erythema migrans (bull-eye pattern), arthritis, myocarditis, peripheral nervous system, facial palsy, meningoencephalitis, chronic fatigue syndrome; Autoimmune and allergic diseases development	ELISA IgM and IgG, western blot IgM and IgG for B. burgdorferi adopted in LIM-17 HCFMUSP standardization	Ceftriaxone intravenously for severe features. Doxycycline or amoxicillin for mild features
Garin-Bujadoux-Bannwarth syndrome	*B. garinii*	Tick-borne (I. scapularis) in Europe.	Painful radiculitis, peripheral motor deficits, lymphocytic meningoencephalitis, and facial palsy	Increase of lymphocytes and protein in CSF. ELISA IgM and IgG for B. burgdoferi, western blot IgM and IgG for B. burgdorferi (lack of standardization)	Ceftriaxone intravenously
Acrodermatitis chronica atrophicans	*B. afzelii*	Tick-borne (I. scapularis) in Europe	Extensive skin atrophy with a shiny appearance or “cigarette paper skin”; Peripheral neuropathy and/or arthropathy	ELISA IgM and IgG for B. burgdorferi, western blot IgM and IgG for B. burgdorferi (lack of standardization)	Doxycycline or amoxicillin or ceftriaxone.

*A. cajennense* Amblyomma cajennense, *B. afzelli* Borrelia afzelii, *B. burgdorferi* Borrelia burgdorferi, *B. garinii* Borrelia garinii, *Baggio-Yoshinari syndrome¶* also known as Brazilian Lyme disease-like illness, *CNS* central nervous system, *CSF* cerebrospinal fluid, *D. nitens* Dermacentor nitens, *LIM-17 HCFMUSP* Laboratórios de Investigação Médica 17 Hospital das Clínicas da Faculdade de Medicina da Universidade de São Paulo, *PAS* periodic acid-Schiff, *PCR* polymerase chain reaction, *ELISA* enzyme-linked immunosorbent assay, *I. scapularis* Ixodes scapularis, *I. pacificus* Ixodes pacificus, *IgG* immunoglobulin G, *IgM* immunoglobulin M, *R. sanguineus* Rhipicephalus sanguineus, *T. whipplei* Tropheryma whipplei

## Data Availability

Data sharing is not applicable to this article as no datasets were generated or analyzed during the current study.

## Abbreviations

ASO: Antisense Oligonucleotides
ANS: National Agency of Supplementary Health
CAR: Chimeric Antigen Receptor
CONITEC: National Committee for Health Technology Incorporation
CNO: Chronic Non-Bacterial Osteomyelitis
CRMO: Chronic Recurrent Multifocal Osteomyelitis
DNA: Deoxyribonucleic Acid
ERT: Enzyme Replacement Therapies
EURORDIS: European Organization for Rare Diseases
FDA: Food and Drug Administration
IgG4-RD: Immunoglobulin IgG4-Related Disease
IEI: Inborn Errors of Immunity
ILD: Interstitial Lung Diseases
IL-1β: Interleukin-1 Beta
mAb: Monoclonal Antibodies
NORD: National Organization for Rare Disorders
NIH: National Institutes of Health
OD: Orphan Diseases
OMIM: Online Mendelian Inheritance in Man
PIRD: Primary Immune Regulatory Disorders
RARAS-BRDN: Brazilian Rare Diseases Network
RD: Rare Diseases
RDC: Rare Diseases Committee
RNA: Ribonucleic Acid
SLE: Systemic Lupus Erythematosus
SCID: Severe Combined Immunodeficiency
siRNA: Small Interfering Ribonucleic Acid
TTR: Transthyretin
UBA1: Ubiquitin Like Modifier Activating Enzyme 1
UDN: Undiagnosed Diseases Network
WES: Whole Exome Sequencing
WGS: Whole Genome Sequencing
WHO: World Health Organization
WB-MR: Whole-Body Magnetic Resonance
